# Improved Classification Approach for Fruits and Vegetables Freshness Based on Deep Learning

**DOI:** 10.3390/s22218192

**Published:** 2022-10-26

**Authors:** Mukhriddin Mukhiddinov, Azamjon Muminov, Jinsoo Cho

**Affiliations:** Department of Computer Engineering, Gachon University, Seongnam 13120, Korea

**Keywords:** fruit classification, fruit and vegetable freshness, YOLOv4, computer vision, object detection, deep learning, convolutional neural network

## Abstract

Classification of fruit and vegetable freshness plays an essential role in the food industry. Freshness is a fundamental measure of fruit and vegetable quality that directly affects the physical health and purchasing motivation of consumers. In addition, it is a significant determinant of market price; thus, it is imperative to study the freshness of fruits and vegetables. Owing to similarities in color, texture, and external environmental changes, such as shadows, lighting, and complex backgrounds, the automatic recognition and classification of fruits and vegetables using machine vision is challenging. This study presents a deep-learning system for multiclass fruit and vegetable categorization based on an improved YOLOv4 model that first recognizes the object type in an image before classifying it into one of two categories: fresh or rotten. The proposed system involves the development of an optimized YOLOv4 model, creating an image dataset of fruits and vegetables, data argumentation, and performance evaluation. Furthermore, the backbone of the proposed model was enhanced using the Mish activation function for more precise and rapid detection. Compared with the previous YOLO series, a complete experimental evaluation of the proposed method can obtain a higher average precision than the original YOLOv4 and YOLOv3 with 50.4%, 49.3%, and 41.7%, respectively. The proposed system has outstanding prospects for the construction of an autonomous and real-time fruit and vegetable classification system for the food industry and marketplaces and can also help visually impaired people to choose fresh food and avoid food poisoning.

## 1. Introduction

Computer vision has numerous benefits in the fruit and vegetable processing industry, enabling automation of numerous activities. Classification and gradation of fruit and vegetable freshness are crucial for the industry manufacturing of highest-quality raw fruits sold in the market. The relevance of fruit safety to the agricultural sector of the global economy is significant. Recently, it has been observed that fruits are sensitive to several infections. This has resulted in global economic pressure in the agricultural industry. The time-consuming manual sorting of many types of fruits and vegetables to assess the quality of fresh and rotting fruits can be minimized by using automatic classification approaches. Therefore, automatic assessment of the quality of fruits and vegetables that enables faster processing of high-quality foods is a rapidly expanding topic of research. Studies have been conducted on using deep neural networks and convolutional neural networks (CNNs) to identify the freshness of fruits and vegetables. Instead of applying typical CNN architectures, this study explores the possibility of transfer learning regarding CNN models for the quality categorization of fruits and vegetables [1].

Fruit classification technology primarily incorporates data from several domains, such as pattern recognition and object classification, to produce a feature set of fruits, from which fruits are categorized through training and learning. Most studies on fruit categorization concentrate on a single problem, with a few exceptions, focusing on multifruit classification [2]. The identification of multifruit categorization has considerable practical application value. For instance, multifruit recognition technology is employed in self-service fruit purchasing in supermarkets of developed countries. In the production line, it may also eliminate human picking mistakes and boost production efficiency. In smart agriculture, multifruit categorization can facilitate the breeding of fruit trees in multivariety mixed orchards and autonomous fruit picking. Furthermore, blind and visually impaired (BVI) people must determine whether fruits and vegetables are fresh or rotten in their daily lives. The conventional research evidence indicates that when fruit spoils, it undergoes a series of biochemical transformations that result in changes in its physical conditions and visual features, such as color and shape, from which the majority of these features can be extracted. A computer vision-based approach is thought to be the most cost-effective solution. Colonizing and generated lesions due to microbe dissemination are frequently observed, and infestation is a primary reason for the spoilage of postharvest fruits [3].

Over the past decade, computer vision communities have focused on recognizing, classifying, and sorting based on external features and counting fruits and vegetables. Studies on fresh and rotten fruit and vegetable classification utilize several approaches, such as support vector machines (SVM), regression trees, Fisher linear discriminant analysis, and k-nearest neighbors (k-NN) to improve the classification accuracy and speed. In a previous study [1], various fruits and vegetables were detected and categorized using machine-learning technologies. A dataset of 15 diverse fruits and vegetables was gathered at different periods and days to reflect real-world situations. One of the limitations of the dataset is the same plain background. Deep-learning (DL) techniques and CNNs have achieved remarkable success in object detection and recognition [4,5] owing to the rapid advances in DL and CNN in recent years. Using a mix of CNN and SVM, Dias et al. [6] extracted features of apple blossoms from a complicated background, with a decent performance of 0.822 F1-score. In recent years, region-based convolutional neural networks (R-CNNs) have gained immense popularity for object detection. R-CNN uses a selective search to generate areas of interest and then regresses the bounding box location with categorization. Zhu et al. [7] propose a mobile visual-based system to evaluate banana grading using two-layer machine learning systems on edge devices and cloud servers. Precisely, the proposed system receives images of bananas on rolling conveyors. In the first layer, SVM classifies bananas based on an extracted feature vector composed of texture and color features. In the second layer, the YOLOv3 model locates the peel’s defected area and decides if the inputs belong to the mid-ripened or well-ripened class. In another study, faster R-CNN [8] enhanced its performance by adding a technique for identifying regions in place of a selective search. However, the classification of fruits as fresh or rotten by blind and visually impaired individuals has not been sufficiently studied.

Despite the aforementioned developments, accurate fruit and vegetable categorization that can assist in choosing fresh products in real-life environments, including supermarkets and homes, remains a great challenge. Existing methods either provide insufficient accuracy [1,2] or are based on a simple plain background with a single object, little occlusion, and stable lighting conditions [4]. Ukwuoma et al. [9] thoroughly discussed the datasets used by many researchers, the clinical descriptors, the model’s implementation, and the challenges of using deep learning to detect and categorize fruits. The results of various deep learning methods used in previous studies to detect and classify fruits are summarized. The goal of this study is to develop a robust and accurate fruit and vegetable categorization system that can assist in choosing fresh products in real-life environments. To this end, a comprehensive dataset was collected from Kaggle, Google, and Bing Images for five varieties of fruits and vegetables with fresh and rotten conditions, as well as multiple objects with complex backgrounds and under various lighting conditions [10]. The dataset currently contains 12,000 images of 20 class fruits and vegetables (as of 2022.09.10), and it is constantly updated with images of new fruits and vegetables as soon as the authors have access to them. The reader is encouraged to download the most recent version of the dataset from the addresses listed above. Furthermore, the well-known YOLOv4 [11] was improved with a Mish activation network and residual network to improve the classification performance. Performance evaluations for fruit and vegetable categorization were then conducted to compare the performance of the proposed deep-learning model with those of state-of-the-art models.

The contributions of this work are summarized as follows:

- An automatic fruit and vegetable classification system was proposed to determine whether the fruits and vegetables are fresh or rotten.
- The proposed classification system first recognizes fruits, such as apples, bananas, oranges, strawberries, and mangoes, and vegetables, including potatoes, tomatoes, carrots, bell peppers, and cucumbers. They are then categorized into the fresh or rotten classes.
- A large fruit and vegetable image dataset that consisted of five types of fruits (apple, banana, orange, strawberry, and mango) and five types of vegetables (carrot, potato, tomato, bell pepper, and cucumber) under various real-life and lighting conditions [10] was gathered and analyzed. It must be noted that the features of the images were trained using large datasets for robust classification of the target object while bypassing overfitting.
- For data enhancement, the automatic movement of labeled bounding boxes method was implemented to rotate the fruit and vegetable images.
- To further enhance the precision of YOLOv4, the activation function was changed to Mish and spatial pyramid pooling and path-aggregation networks are adopted. The experimental results show that the proposed system and dataset achieve robust performance compared to other state-of-the-art approaches (YOLOv3, YOLOv4, and their tiny versions).
- Lastly, a mobile application was developed to demonstrate real-time performance for blind and visually impaired people.

The remainder of this paper is organized as follows. Section 2 reviews the literature on fruit and vegetable classification approaches. The data-collection process and data augmentation are described in Section 3. Section 4 and Section 5 explore the proposed fruit and vegetable classification method and discuss the experimental results and analysis, respectively. Section 6 outlines our findings and proposes directions for future research.

## 2. Related Work

Research on fruit and vegetable classification and categorization using cutting-edge deep learning has significantly improved. For example, faster region-based CNN techniques have been applied for multiclass fruit recognition in harvesting, intelligent farming, and packaging sectors, as mentioned in two previous studies [12,13]. In these studies, the networks were trained using outdoor orchard photos in real-life situations, such as at different times of the day and under diverse lighting conditions, to achieve better performance. Another method for strengthening the mask R-CNN architecture is to add a suppression branch to reduce erroneous detections caused by occlusion, thereby increasing the accuracy and robustness of apple detection in orchards [14,15].

Many attempts have been made for fruit recognition and classification in robot harvesting and farming using the deep learning approach [16,17,18]. A previous study [19] proposed an improved MobileNetv2 with ImageNet weights and fine-tuning by freezing the first 130 layers of MobileNetV2 and training the remaining 25 layers for fruit classification. They obtained real-time performance using a 13MP AR1335 camera connected to an NVidia Jetson Xavier and achieved 97% accuracy in the fruit classification of six classes: fresh/rotten apples, fresh/rotten bananas, and fresh/rotten oranges. Kazi et al. [1] implemented and tested various architectures of classical CNNs and a residual CNN, such as AlexNet, ResNet50, and VGG-16, for fruit classification. The dataset used in a previous study [1] consisted of six classes, similar to that in another study [20]. The experimental results showed the ResNet50 and AlexNet models could be used to identify the rottenness of other perishable goods with higher than 99% accuracy on the given dataset; they have the potential to be used for determining the freshness of other fruits and vegetables at an industrial level [1]. Alam et al. [4] reviewed freshness sensors as smart packaging technologies for monitoring fruit quality. The biology of fruits, their classifications, growth, and different stages of processing and harvesting were discussed owing to the need for smart packaging that could help reduce fruit waste during the harvesting, post harvesting, and packaging stages. Chen et al. [20] introduced a classification method that identified the external quality of fruits by using an improved YOLOv3 model. The experimental results show that the proposed application achieves an accuracy rate of up to 88% after testing on 6000 images of fruits, such as apples, oranges, and lemons. Ni et al. [21] analyzed the changes in freshness of bananas and strawberries using the GoogLeNet model as the extractor and the AlexNet and VGGNet models as the classifier. The results showed that the model could detect the freshness of bananas with an accuracy of 98.92%, which was higher than the human detection level. 

Fruit classification methods based on deep learning are widely used in the postharvesting stage and fruit industry. Fan et al. [22] proposed a post harvesting quality sorting and grading method that sorted apples into normal and defective apples. The dataset consisted of 300 Fuji apples with normal surfaces and various types of defects, such as physical or insect damage, rottenness, and scarring. The CNN-based model was loaded into the custom software of the fruit sorting system to validate its online performance using 200 independent apples, obtaining an accuracy of 92% with a processing time per apple of less than 72 ms. Roy et al. [17] improved the UNet model for the detection of rotten or fresh apples based on defects present in the peel of the fruit. A total of 4035 apple images, including 1693 fresh apples and 2342 rotten apples, were used for training the modified UNet model. The modified UNet model generated enhanced outputs compared to those obtained by the original UNet; the training and validation accuracies of the original and modified UNet models were 97.46% and 97.54%, respectively. Bhargava et al. [23] implemented an apple fruit quality evaluation system that preprocesses the image and segments the defected part by the grab-cut method and fuzzy c-means clustering to segment six different varieties of apples, such as Fuji, York, Golden Delicious, Red Delicious, Granny Smith, and Jonagold. The classification of fresh and rotten apples is done by utilizing logistic regression, SVM, sparse representation classifier, and k-NN classifiers. Palakodati et al. [24] proposed a CNN model to achieve high accuracy in the classification of fresh and rotten fruits, such as apples, bananas, and oranges. The total size of the dataset was 5989 images. The training set contained 3596 images, the validation set contained 596 images, and the test set contained 1797 images belonging to six classes. 

However, in the majority of the studies [18,19,20,21,22,23,24], the dataset consisted of a single fruit species under identical illumination conditions, rendering the conclusions less convincing. A further drawback of the existing datasets is that the vast majority of them contain only a small number of fruit types and no vegetable varieties. In this study, a comprehensive fruit and vegetable database containing several species of fruits and vegetables under different lighting conditions was employed. In addition, earlier research has been confined to categorizing only fruits, and the quality evaluation and sorting of vegetables has not been adequately investigated. Consequently, an improved YOLOv4 model was designed with improved performance and classification of fruits and vegetables compared with that of the abovementioned methods.

## 3. Data Collection and Processing

**Figure 1:**
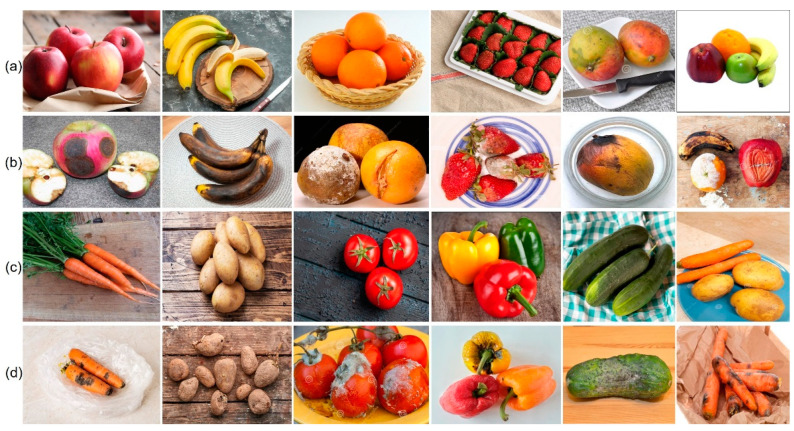
Sample images of fruit and vegetable dataset with multiple objects and various backgrounds: (**a**) fresh fruits, (**b**) rotten fruits, (**c**) fresh vegetables, (**d**) rotten vegetables.

This study selected the most popular varieties of fruit and vegetables to categorize their quality. Images of five fruits (apple, banana, orange, strawberry, and mango) and five vegetables (carrot, potato, tomato, cucumber, and bell pepper) were obtained from Kaggle, Google, and Bing images, and using a mobile camera. The proposed fruit and vegetable dataset contained 12,000 images in total. Each type of fruit and vegetable was divided into fresh and rotten classes and consisted of 20 classes. To decrease false categorization, each class comprised approximately 600 photos with diverse lighting conditions, such as back lighting, front lighting, dispersed lighting, and side lighting. Figure 1 shows some sample fruit images from the dataset. The similarity between the two freshness categories is high for the same fruits and vegetables. Moreover, different fruits and vegetables may seem identical owing to their form and color, yet the same class may appear differently in multiple cases, making this a challenging dataset. The dataset for categorizing fruits and vegetables is accessible to the public for future research. The fruit and vegetable freshness datasets are presented in Table 1. The proposed dataset included 12,000 original images separated into five categories of fruits and vegetables. Digital pictures with dimensions of 2160 × 2160 were acquired using Samsung Galaxy S8 mobile cameras and gathered from different online sources, such as Fruit360 [25] and Sriram R.K. [26], which provided samples of the pure-fresh category and a single item with a white background, respectively. RGB (Red, Green, Blue) pictures were gathered in dark and bright lighting settings, with varied scene complexities ranging from a simple white background to complex backgrounds with shifting color patterns. Images comprised single and multiple items, with 70% of the sample consisting of single objects and the remainder including multiple objects ranging from 2 to 6, as shown in Figure 1. The entire dataset was annotated manually using the LabelImg tool 1.8.0.

Following the collection of 12,000 images, the original images were scaled, organized, and categorized into formats that could be used to train and classify fruits and vegetables using the proposed deep-learning model. Obtaining a significant amount of labeled training data is an essential success component for every deep-learning model [27]. However, obtaining effective fruit and vegetable classification results using this dataset in real-world contexts proved difficult. This can be attributed to overfitting, underfitting, or a class imbalance. An overfitting model cannot capture picture patterns accurately. Because a lack of data might cause underfitting, the image data augmentation approach (changing and reusing pictures) to increase the predictive capability of the model. The LabelImg tool version 1.8.0 was utilized to rectangle annotate the images in accordance with the YOLOv4 training annotation. The 12,000 annotated images were separated into training, validation, and test sets, with 80% designated for training, 10% for validation, and 10% for testing.

In addition, as shown in Table 2, the current fruit and vegetable classification datasets are evaluated and compared to evaluate freshness categories, overall size, and number of classes. Fruits and vegetables were only found in the fresh category in many publicly available databases, with no rotting category. Furthermore, each dataset contained only the images of a single fruit or vegetable. A dataset encompassing diverse fruits and vegetables, as well as the two categories of fresh and rotten, is required for the robust classification of fruits and vegetables.

### Data Augmentation

It is crucial to make the fruit and vegetable classification system more resistant to varied conditions by including various fruits and vegetables in the dataset. However, the dataset may contain unavoidable biases that are not readily apparent to the researcher, which may lead to overfitting of the training dataset. To define this potential risk, it is assumed that additional information can be recovered from the training dataset if the pictures are altered in various ways. This is known as data augmentation and may replicate a broader representation of images of fruits and vegetables, preventing probable overfitting to the training dataset [36]. How does one determine which data augmentation approach to employ?

**Figure 2:**
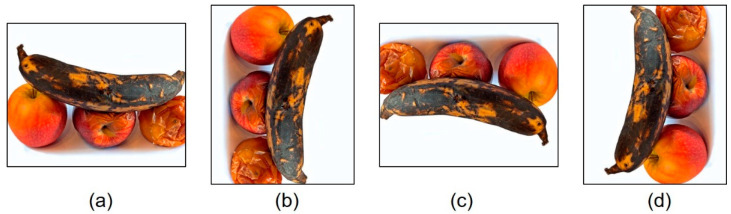
Image data augmentation using geometric transformations: (**a**) original image, (**b**) 90° rotation, (**c**) 180° rotation, (**d**) 270° rotation.

There are two setups of data augmentation: pixel level and spatial level. Pixel-level adjustments alter the pictures while leaving the bounding boxes untouched. Some examples include blurring, adjusting the brightness or exposure, adding noise, CutMix, Cutout, and other pixel-level alterations. This is important if the researcher expects to maintain the bounding boxes and avoid distorting the form of the target object. In contrast, spatial-level transformations affect both the notion and bounding box, making the transformation significantly more difficult to implement than pixel-level transformations. However, spatial-level changes have proven to be more successful in increasing the performance of object recognition and detection approaches [37]. Both setups were used in this study. After reviewing [38,39] and conducting [40,41] tests, our study determined that the image–data augmentation approaches based on spatial-level modifications, such as rotation and mosaic image enhancement, were the most successful. The capabilities of the CNN models were determined by the size and resolution of the picture datasets used for training. As shown in Figure 2, the number of images was expanded in the dataset to classify fruits and vegetables by rotating each original image by 90°, 180°, and 270°. Consequently, the existing training images are updated to make them applicable to a wider variety of contexts, enabling the model to learn from a broader set of scenarios.

Manually rotating and categorizing all images in the dataset takes a long time to complete. Software was developed to automatically rotate images using the OpenCV library to automate the image editing process. The images were resized to the dimensions of 416 × 416, 512 × 512, and 608 × 608. Black padding was used to prevent the alteration of the aspect ratio of the fruit and vegetable images. The dataset was enlarged from 12,000 to 43,667 images by augmentation, as shown in Table 3.

The better the image quality, the less necessary is the feature lost [42]. The improved YOLOv4 [11] was used to categorize fruits and vegetables as fresh or rotten. Mosaic data augmentation was employed for image enhancement in the YOLOv4 model. Mosaic refers to the CutMix data enhancement approach that stitches many images together. Mosaic employs four images for stitching to enhance the background of the classified object. The data from the four images may be computed immediately during batch normalization. The data improvement procedure is as follows: read four images at once; then, flip, zoom, and adjust the color spectrum of the four images; create a mix of images and anchors.

## 4. Proposed Method

This section discusses the creation of the proposed deep learning-based fruit and vegetable categorization solution, which systematically blends a deep neural network backbone with spatial pyramid pooling (SPP), feature pyramid networks (FPN), and path aggregation network (PAN) modules. The proposed system can be thought of as a combination of researchers who work in object, fruit detection and assistive technologies for visually impaired areas. After having applied the artificial intelligence (AI) approaches, namely deep learning (DL) and transfer learning (TL) networks, to our research, fruit and vegetable classification performance was improved to promote healthy eating for BVI people and reduce the misclassification of fresh and rotten fruit in agricultural industries. The idea of TL is introduced into the research of fruit and vegetable classification based on a custom dataset, and the optimized YOLOv4 model with minor improvements is proposed. The original YOLOv4 pre-trained on the COCO dataset with 80 classes is used as the backbone framework for fruit and vegetable classification. 

### 4.1. Modular Representation of the Proposed Fruit and Vegetable Classification System

**Figure 3:**
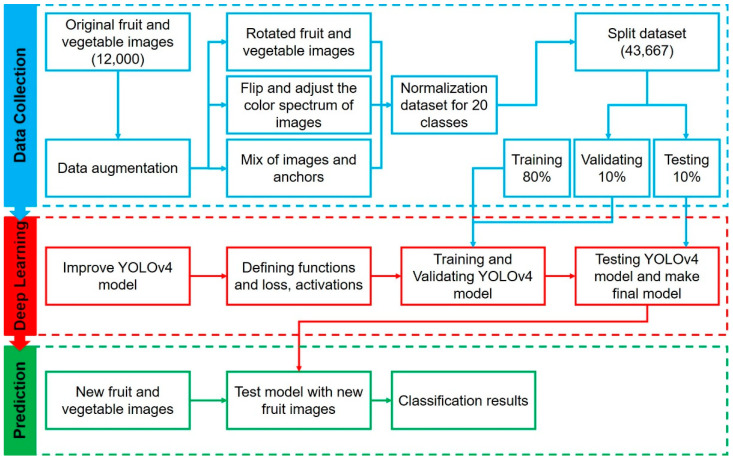
Modular representation of fruit and vegetable classification system.

The modular representation of the proposed determining freshness of fruit and vegetable approach is shown in Figure 3. The first step includes data collection and processing for the training model. In the second step, our study defined a deep learning model to classify fruit and vegetable by training and testing iteratively. Subsequently, the prediction step can achieve the final result for the classification of fresh and rotten fruits.

The YOLOv4 model is an improved version of YOLOv3. The Darknet-53 backbone in YOLOv3 was replaced with the CSPDarknet-53 backbone in the YOLOv4 model. The value is generated by the last residual network structure in CSPDarknet-53. The CSPDarknet-53 classifier uses the Mish activation function for training to increase the classifier and detector accuracy by adjusting the pre-training weight of the classifier. Thus, CSPDarknet-53 is more appropriate for object classifiers and detectors. 

### 4.2. Block Diagram of Improved YOLOv4 Model

**Figure 4:**
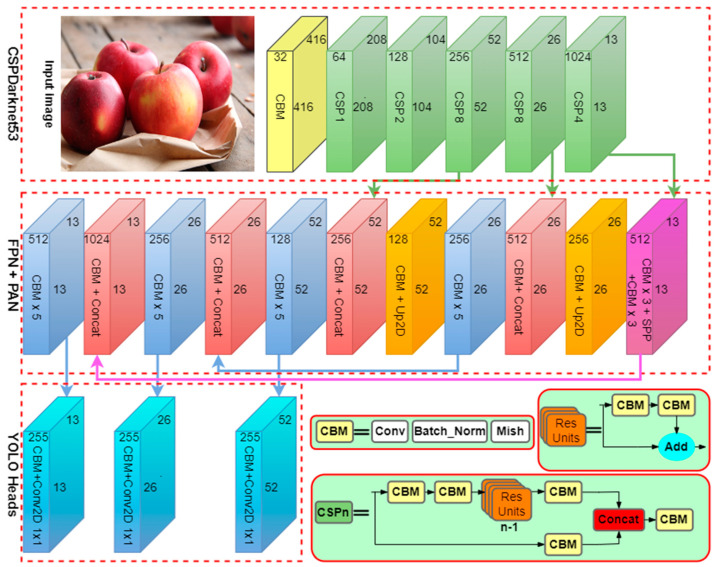
Block diagram of the proposed fruit and vegetable categorization using optimized YOLOv4 model.

The YOLOv4 model is separated into three sections: the CSPDarknet-53, neck, and head. The backbone section of the model is the CSPDarknet-53 network. The neck section of the model is composed of SPP, FPN, and PAN networks, anticipating more promising use of the feature extracted by the backbone. The head section is a prediction that uses the previously extracted features and outputs the final categorization result [43]. As shown in Figure 4, the flow chart of the fruit and vegetable categorization is based on the improved YOLOv4 model. The categorization procedure is as follows:

1. A fruit and vegetable image is input into the deep-learning network.
2. The backbone section and Mish activation function are employed to extract information from the image.
3. The neck section comprises the SPP, FPN, and PAN modules, which are used to ensure more efficient use of the extracted characteristics from the backbone.
4. The prediction section employs previously extracted characteristics to provide the final detection result.

We then discuss the contents of individual sections. The CSPDarknet-53 structure serves as the backbone, with five cross-stage partial connection (CSP) networks (green cuboid), and each CSP includes multiple convolutional, batch normalization and Mish (CBM) networks. The CBM network is a convolution procedure that uses batch normalization and Mish activation functions. The CBM module is an essential component of the CSP network. Leaky ReLU and Mish activation functions are experimentally tested in neck and head sections of YOLOv4 and obtained with superior precision using Mish activation. However, the network training time was increased when Mish activation function was used.

### 4.3. Activatiopn and Loss Functions of Improved YOLOv4 Model

Researchers require an activation function to establish nonlinear mappings between inputs and outputs to gain access to a significantly richer view space that benefits from deep representation. The leaky ReLU is a common activation function in deep learning; however, Mish performs better on average than leaky ReLU. Utilizing Mish is a crucial advancement that can increase the classification accuracy. The network adopts Mish activation function over the backbone, neck, and head sections. The Mish activation function is calculated as follows:

(1) $$y_{mish}=x\;\tan h(\ln\left(1+e^{x}\right)),$$

In the following Equation (2) represents leaky ReLU function:

(2) $$y_{leaky\;relu}=\{\begin{array}{cc} x, & if\;x\geq 0\\ \lambda x, & if\;x<0 \end{array}$$

One of the advancements in network technology is the use of the CSP module. In this module, CSP is used to represent n residual units. The CSP module structure is presented in Figure 4, where the “Add” operation adds tensors without extending the dimensions, whereas the Concat action adds tensors and dimensions.

The improved section of YOLOv4 is as follows:

- The backbone is updated from CSPDarknetConv and CBL to CSPDarknetConv and CBM by adjusting the activation function.
- The residual block structure is updated to split the residual network, with one portion stacking the residual network and the other acting as the residual edge. It proceeds directly to the end, with only minor processing. This section avoids various leftover structures, generally known as the CSP module.
- The model adopts the SPP and PAN modules.

The YOLO model converts the detection task into a regression task and produces the boundary coordinates and probabilities of each class. The YOLOv4 loss functions comprising bounding box location loss ($L_{location}$), confidence loss ($L_{confidence}$), and classification loss ($L_{class}$) [6] are applied to train the network to achieve object detection and recognition based on an artificially defined area if the center of the observed object falls inside the grid.

(3) $$Loss=L_{location}+L_{confidence}+L_{class}$$

(4) $$Location=IoU-\frac{c^{2}\left(b,b^{af}\right)}{d^{2}}-\alpha v,$$

where $c^{2}\left(b,b^{af}\right)$ denotes the Euclidean distance between the center points of the prediction frame and actual frame and *d* denotes the diagonal distance of the minimum required area that can contain both the prediction frame and the real frame:

(5) $$\alpha =\frac{v}{1-IoU+v}$$

(6) $$v=\frac{4}{\pi^{2}}{\left(arctan\frac{w^{af}}{h^{af}}+arctan\frac{w}{h}\right)}^{2}$$

(7) $$L_{location}=1-IoU+\frac{c^{2}\left(b,b^{af}\right)}{d^{2}}+\alpha v,$$

where *IoU* is determines the precision of object detection and displays the intersection ratio between the predicted bounding box and ground truth bounding box. YOLOv4 can detect and recognize objects with complex backgrounds and high similarity and is appropriate for fruit and vegetable classification

## 5. Experimental Results and Analysis

The experimental setup and results of the fruit and vegetable categorization model are described in this section. The proposed deep CNN and other alternative models were trained on a PC with an 8-core 3.70 GHz CPU, 32 GB RAM, and NVidia GeForce 1080Ti GPUs. For training and testing, fruit and vegetable datasets were used. The following are the important settings for the training experiments: batch size of 32 pixels, input image size of 416 × 416, learning rate of 0.001, and subdivision of 8. To reliably and accurately classify fruits and vegetables, researchers must examine the classification performance. This work analyzes and compares several object detections, such as improved YOLOv4, YOLOv3, YOLOv3-tiny, YOLOv4, and YOLOv4-tiny, to train and test fruits and vegetables and classification models. Experiments show that YOLOv4 has a higher precision and training speed than YOLOv3 and YOLOv3-tiny and that improved YOLOv4 accurately classifies and categorizes more fruits and vegetables as fresh or rotten than other models. The results demonstrate that the enhanced YOLOv4 model accurately classifies fruits and vegetables as fresh or rotting. The experimental evaluations were determined by qualitative and quantitative evaluations.

### 5.1. Qualitative Evaluation

**Figure 5:**
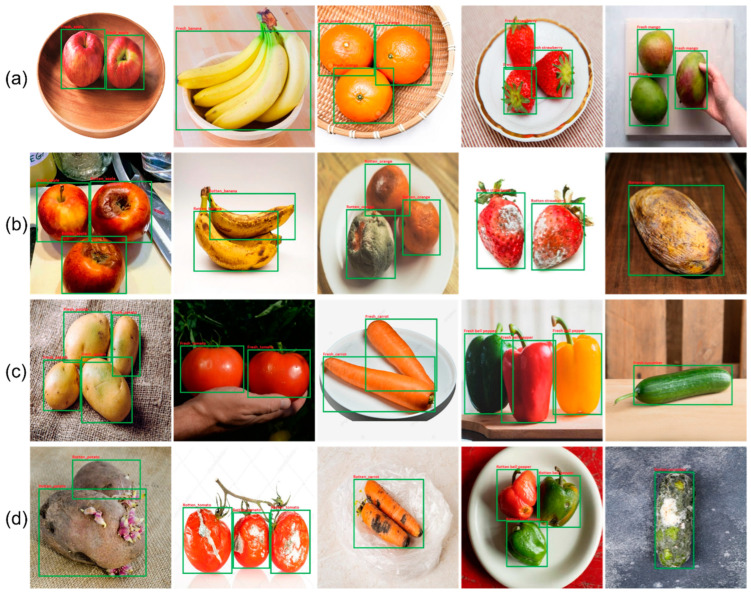
Classification results of the proposed fruit and vegetable model. (**a**) Fresh fruits, (**b**) Rotten fruits, (**c**) Fresh vegetables, (**d**) Rotten vegetables.

First, a qualitative evaluation of the proposed fruit and vegetable categorization model was conducted. Thus, six pictures of fresh fruits and vegetables and six pictures of rotten fruits and vegetables were selected from our test set. These 12 pictures depict various situations and circumstances, including multiple fruits and vegetables in a fresh or rotten state, with the rot produced by bacteria, yeast, and molds. Figure 5 illustrates the qualitative results of the improved YOLOv4 model for 12 pictures.

As shown, the proposed fruit classification utilizing the YOLOv4 model correctly classifies the fresh and rotten fruits under various conditions. It can be integrated as a programmable module into smart glasses [41] to assist blind and visually impaired users in identifying fresh or rotten fruits and vegetables in their environment.

**Figure 6:**
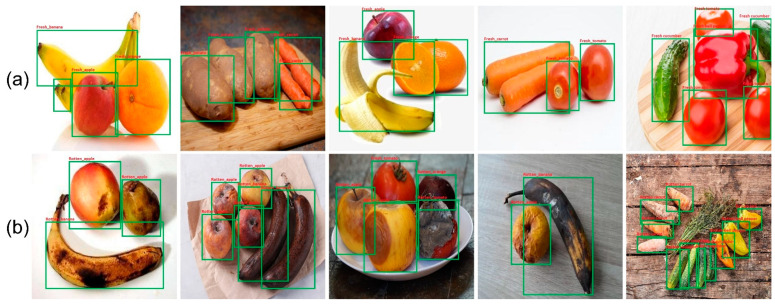
Classification results of the proposed fruit and vegetable model for multiple objects in single image: (**a**) fresh fruits and vegetables, (**b**) rotten fruits and vegetables.

Furthermore, the proposed technique was tested by using multiple objects in a single picture to ensure its strength and trustworthiness. Accurately assessing the freshness of fruits and vegetables is critical to avoiding food poisoning and other inconveniences. In some circumstances, fruits can also be found with other species. Therefore, it is essential to distinguish them accurately from other fruits and vegetables. Figure 6 shows several instances of fruit categorization for multitype fruits and vegetables in a single picture.

### 5.2. Quantitative Evaluation

Quantitative assessment measures were utilized in this study to assess the fruit and vegetable categorization systems. It evaluates the trained model using precision, recall, and average precision (AP) to determine a suitable threshold for the model and then selects the appropriate parameters using the confidence coefficient of the model prediction.

Similar to that in prior studies [41,44], quantitative tests were conducted and examined the findings using object detection assessment measures, such as precision, recall, and AP. Precision is the classifier capacity to identify only the relevant items, that is, the fraction of true positives recognized. The fraction of true positives found among all ground truths evaluates the ability of the model to identify all relevant situations. A good model can recognize most ground-truth items (high recall) while recognizing only the relevant objects (it exhibits high precision). The false-negative value of the perfect model is 0, while the false-positive value is 0. Precision and recall metrics were computed by comparing the results of the proposed approach with pixel-level ground-truth pictures. The following equations were used to calculate the precision and recall metrics of the fruit and vegetable classification system:

(8) $$Precision_{C_{ij}}=\frac{TP_{C_{ij}}}{TP_{C_{ij}}+FP_{C_{ij}}},$$

(9) $$Recall_{C_{ij}}=\frac{TP_{C_{ij}}}{TP_{C_{ij}}+FN_{C_{ij}}},$$

where *TP* represents the actual number of true positive samples, *FP* represents the number of false positive samples, *FN* represents the number of false negative samples, and *C* represents the number of categories. *AP* is the region under the precision–recall curve. In general, the higher the AP number, the more accurate is the classifier. The following equation calculates the *AP* value:

(10) $$AP_{C_{ij}}=\frac{1}{m}\sum_{j=1}^{m}Precision_{C_{ij}},$$

Furthermore, detection evaluation metrics such as *AP50*, *AP75*, *APS*, *APM* and *APL* were used from COCO evaluation. Here, *AP50* and *AP75* represent as average precision at *IoU* = 0.5 and 0.75 respectively. In object detection, particularly fruit and vegetable detection, there are various size of objects, such as small, medium, and large. Therefore, researchers used *APS* (the number of pixels in the segmentation mask < $32^{2}$), *APM* (the number of pixels in the segmentation mask between $32^{2}$ and $96^{2}$) and *APL* (the number of pixels in the segmentation mask > $96^{2}$) for performance evaluation. 

### 5.3. Quantitative Evaluation with Initial 12,000 Images

**Figure 7:**
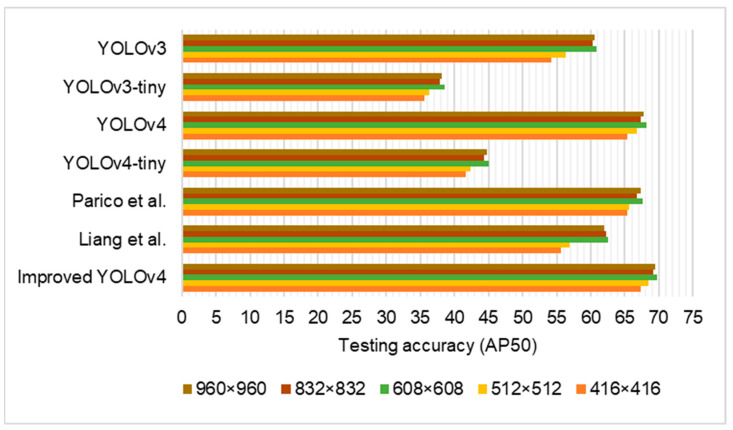
Performance of the models with varied test picture sizes [36,47].

In Figure 7, the performance of the improved YOLOv4 and other series were present by changing the input image size in the dataset. The most robust results were achieved with an image weight and height of 608 × 608 pixels. 

Initially, the deep CNN model was evaluated with the initial 12,000 images and then with the entire augmented dataset with 43,667 images. As shown in Table 4, the deep CNN model performed better with the augmented dataset than with the original dataset. Presumably, data augmentation methods enable the training of objects in various situations and views.

As explained in Section 4, the YOLOv4 model design was modified to obtain more accurate fruit and vegetable categorization results. The Mish activation function was employed to eliminate gradient explosion, which reduced the running time and increased the strength of the deep CNN model. The performance of the proposed method was evaluated by comparing the final precision of several variants of YOLO on the original fruit and vegetable image collection (12,000 images) (Table 5).

**Table 5:** Comparison of fruit and vegetable classification models training precision with original fruit and vegetable images.

Models	Training Image Size	Training Results (*AP50*)	Testing Image Size	Testing Result (*AP50*)	Training Time	Iteration Number
YOLOv3 [45]	416 × 416	63.7%	608 × 608	60.8%	82 h	225
YOLOv3-tiny [45]		43.4%		37.8%	11 h	
YOLOv4 [11]		71.3%		68.2%	71 h	
YOLOv4-tiny [11]		48.6%		45.1%	8 h	
Parico et al. [36]		70.7%		67.6%	72 h	
Fu et al. [46]	224 × 224	62.4%		58.5%	42 h	
Liang et al. [47]	416 × 416	64.3%		62.6%	80 h	
Improved YOLOv4		72.5%		69.2%	68 h	

### 5.4. Quantitative Evaluation with Augmented 43,667 Images

Furthermore, the performance of the proposed method was evaluated by comparing the AP results of several versions of YOLO on the enhanced fruit and vegetable dataset (43,667 images). The results presented in Table 6 demonstrate that the improved YOLOv4 model places first in the training and testing phases, with precision of 75.8% and 73.5%, respectively. YOLOv4 obtained a precision of 72.6% (a difference of 0.9% from the enhanced YOLOv4 model) in testing, slightly behind the improved YOLOv4 model in terms of testing precision. During training, YOLOv4-tiny and YOLOv3-tiny achieved training precisions of 53.6% and 46.2%, respectively. Because of the significant number of dataset images, these models required more time to train than those used in earlier evaluations. Despite having a longer processing time than the YOLOv4-tiny approach, YOLOv4 was considered the most effective, robust fruit and vegetable classification model, with the best prediction precision. The training precision of the improved YOLOv4 model was increased from 72.5% to 75.8% (3.3%) and the test precision from 69.2% to 73.5 % (4.3%) using data augmentation techniques.

Table 7 compares the enhanced YOLOv4 model with other variants of YOLO object detection models. To compare and assess the performance of the object detector models, identical training and testing images were utilized from the custom fruit and vegetable dataset.

According to the AP, AP50, AP75, and APL assessment measures, the enhanced YOLOv4 model exhibited the best fruit and vegetable categorization performance on our image dataset. The proposed technique achieved the second-best overall performance, falling short of the original YOLOv4 in the APS and APM assessment measures.

### 5.5. Confusion Matrix Evaluation

**Figure 8:**
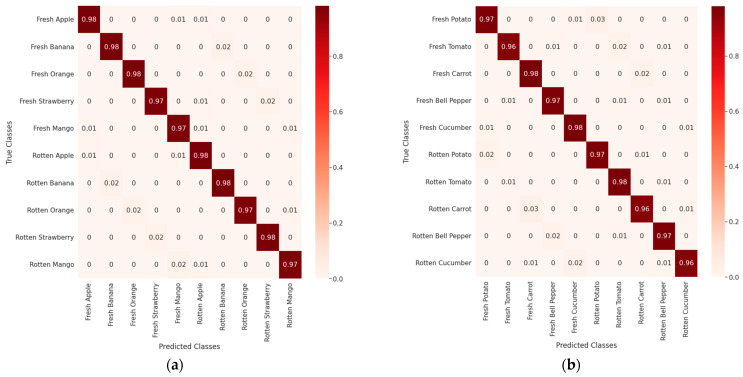
Results of the confusion matrix (**a**,**b**) for the classification of fruits and vegetables, respectively, using the improved YOLOv4.

In addition, the improved YOLOv4 model was evaluated using a confusion matrix for fruit and vegetable classification, as shown in Figure 8. In Figure 8, the left image represents the fresh and rotten fruit classification, whereas the right image represents the fresh and rotten vegetable classification confusion matrix. The authors randomly selected 100 original images from a test set for every 20 classes. Approximately 85% of randomly selected images are single objects with a plain background, whereas the remaining 15% of images are multiple objects with a complex background, as depicted in Figure 1.

The above evaluation results show that the AP score of the proposed method is 50.4%, and the average result of the confusion matrix of fruits is 97.6%. In comparison, the average result of the confusion matrix for vegetables is 97%. Therefore, the improved YOLOv4 model can recognize fruits and vegetables and solve the fruit and vegetable classification problem by categorizing them into fresh or rotten classes. This establishes a foundation for automating the operations of food enterprises and supermarkets and providing customers with fresh fruits and vegetables.

### 5.6. Implementation Environment for Blind and Visually Impaired People

**Figure 9:**
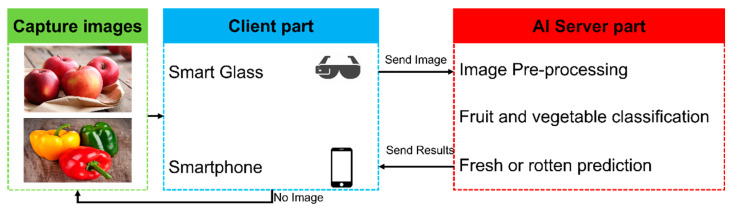
The overall diagram of the client-server architecture for mobile application.

A mobile application was created to implement the proposed fruit and vegetable classification system in real-life situations. Our previous works [37,41] introduced a client-server architecture-based smart glass system for blind and visually impaired people. The trained fruit and vegetable classification model was added for prediction in the AI server part. The working of the client and server architecture is as follows:

- Images are captured using a smartphone camera or smart glass (green and blue boxes in Figure 9).
- The smartphone sends the photos to the AI server for prediction results (blue box in Figure 9).
- The AI server receives the images, processes them, and then predicts the result (red box in Figure 9).
- The AI server converts text results to audio using a text-to-speech model (red box in Figure 9).
- The smartphone gets the final audio result and text prediction (blue box in Figure 9).
- The smartphone reads out the audio result and displays the text prediction (blue box in Figure 9).

The general design and process of client-server architecture are shown in the following Figure 9. The client part consists of a smartphone, and smart glass, while the AI server part consists of a computer and deep learning model. In the client part, the BVI user firstly establishes a Bluetooth connection between a smart glass and a smartphone. Following that, the user can ask the smart glass to capture images, which are then sent to the smartphone. In this scenario, smart glasses’ power consumption can be reduced, which is far more efficient than continuous video scanning. Following that, the AI server’s results are delivered in text and voice feedback via earphones, speakers, or smartphones. In the AI server part: first, received image from client is pre-processed for noise removing. Furthermore, the fruit and vegetable classification model predicts fresh or rotten results. After that, the predicted results are converted to audio format using text-to-speech method and sent to the client part along with text results.

**Figure 10:**
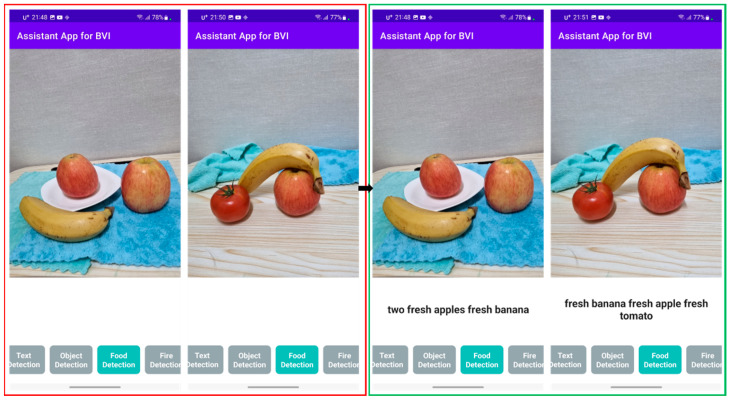
Mobile application of fruit and vegetable classification system for BVI people.

In addition, researchers also tested the mobile demo application with fruit and vegetable examples in real-world scenarios. As shown in Figure 10, input images are in the red box, while corresponding output results are in the green box. The experimental results show the true classification of fresh and rotten fruits. The whole project of assistant application for BVI consists of multiple modules, such as text detection, object detection and fire detection, as explained in our previous works [37,41]. In this paper, the food detection module was explained to determine the freshness of fruit and vegetable. 

### 5.7. Limitation and Disscussion

**Figure 11:**
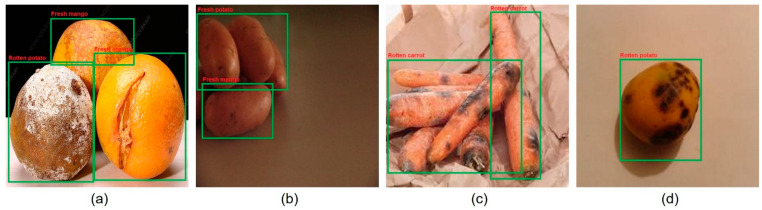
Experimental results showing limitations of the proposed system: (a) Rotten oranges are misclassified as rotten potato, fresh mango, and fresh orange, (b) Fresh potatoes are misclassified as fresh mango, (c) Rotten carrots, (d) Rotten mango is misclassified as rotten potato.

Despite the achievements mentioned above, the proposed fruit and vegetable classification system has shortcomings. These include detecting multiple objects with small sizes and overlapped regions. In addition, some fruit and vegetable external features, such as color, shape, and texture, are very similar. In these cases, the proposed system misclassifies fruits and vegetables. Figure 11 presents these misclassification results. These limitations mainly occur in complex backgrounds and when the shape and color of objects are similar. Figure 11a shows that rotten oranges are classified as fresh mango, the rotten potato, and fresh orange. Fresh potato is classified as fresh mango, while rotten mango is classified as a rotten potato in (a) and (b) columns of Figure 11, respectively. Furthermore, it is also necessary to improve the number of fruit and vegetable classes so BVI users worldwide can differentiate between fresh and rotten daily consumed fruits and vegetables. 

Our next aim is to increase the number of object classes in the dataset and then update the smart-glasses-based system using an RGB-D camera or ultrasound sensor that detects the distance to the object. Adding a method to determine the nutrition of the various food and how far it is from a blind person is also one of the tasks that could expand the scope of this field. This study covered only the AI server part of the wearable assistive fruit and vegetable classification system. A case study with BVI people could not be performed owing to device patenting, the pandemic, and other circumstances. In addition, the current research analysis indicates that it is challenging to classify fruits and foods in different scenarios using vision-based food classification approaches.

Furthermore, the performance of frame processing time for each stage is obtained, including Bluetooth image transmission between smart glass and smartphone, 5G/WiFi image transmission time between smartphone and server, and the deep-learning model’s image processing time in the AI server. The average processing time, in seconds, for each stage is shown in Table 8. As can be seen, the entire process takes a total of 0.859 s, making it practical for use in real-world scenarios.

## 6. Conclusions

This fruit and vegetable categorization was implemented in this study employing deep CNN models and an optimized YOLOv4 object detector. The proposed fruit and vegetable classification system was trained using a collected image dataset of fresh and rotten fruits and vegetables. It categorizes fruits and vegetables as fresh or spoiled to enable automation of the food industry and help blind and visually impaired people perform daily household tasks. A dataset of 12,000 pictures with 20 classes of five types of fruits and vegetables were collected for model training and testing. The qualitative and quantitative performances of the proposed system were compared during the experiments to those of other well-known one-stage object detectors. The experimental and assessment findings demonstrated that the improved YOLOv4 model was accurate and outperformed YOLOv4 on our fruit and vegetable datasets [10] with 73.5% and 72.6% AP, respectively. The proposed fruit categorization approach is efficient and useful in various applications, including the food industry, supermarkets, and assistive technologies for blind people. The following highlights can be summarized based on the experimental results:

1. The optimal deep-learning strategy was identified for determining the freshness of fruit and vegetable classification problems. The architectural properties of the YOLOv4 model and primary classification issues were investigated. In the proposed model, the backbone section extracts more in-depth features of the target objects and decreases the interference of the complex background with the target object. The neck expands the acceptance coverage of the model features with less calculation and extracts more semantic and positioning information of the target object to detect fruit and vegetable regions.
2. Using the improved YOLOv4, the fruit and vegetable classification method can precisely categorize fresh or rotten fruits and vegetables under varying lighting and occlusion situations for different types and classes, providing accurate data for the food industry, supermarkets, and blind people.

In the future, the authors plan to continue to explore new approaches, such as self-supervised and semi-supervised learnings and improving the accuracy of the classification model and image datasets to classify other fruits and vegetables. Furthermore, our plan to work on the hardware component to develop a prototype of the device that can assist BVI people in determining the freshness of fruits and vegetables.

## Figures and Tables

**Table 1:** The fruit and vegetable dataset consists of 12,000 images of 5 fruits and 5 vegetables categorized into 20 classes. Image size is 608 × 608.

Fruit and Vegetable Name	Categories of Freshness	
	Fresh	Rotten
Apple	612	588
Banana	624	576
Orange	609	591
Strawberry	603	596
Mango	605	593
Potato	615	585
Carrot	620	580
Tomato	604	596
Cucumber	608	593
Bell pepper	611	591
Total	6111	5889

**Table 2:** The comparison of current fruit and vegetable dataset for fruit classification.

Datasets	Total Size	Image Size	Classes	Fruit Types	Vegetable Types	Fresh Category	Rotten Category
Fruit 360 [25]	90,483	100 × 100	131	83	48	√	-
Guava fruit [28]	400	520 × 530	5	1	-	√	√
Citrus Fruit [29]	150	256 × 256	5	1	-	√	√
Golden apple [30]	120	320 × 320	3	1	-	√	√
Fruit freshness [31]	6300	224 × 224	14	7	-	√	√
Hussain et al. [32]	44,406	320 × 258	15	10	5	√	-
Papaya fruit [33]	300	227 × 227	3	1	-	√	-
Sriram R.K. [26]	10,901	416 × 416	6	3	-	√	√
Kritik S. [34]	4320	618 × 618	36	10	26	√	-
Tomato [35]	43,843	100 × 100	2	-	1	√	-
Our dataset [10]	12,000	various	20	5	5	√	√

**Table 3:** Distribution of training and testing images in the fruit and vegetable classification dataset.

Fruit and Vegetable Dataset	Training and Validation Images		Test Images	Total
	Original Images	Rotated Images	Original Images	
Fresh fruits	2684	8052	369	11,105
Rotten fruits	2593	7779	351	10,723
Fresh vegetables	2690	8070	368	11,128
Rotten vegetables	2592	7776	353	10,721
Total	10,559	31,677	1441	43,667

**Table 4:** Fruit and vegetable classification model training precision for original (12,000) and augmented images (43,667).

Model	Image Size	Training Precision (*AP50*)		Time for Model Training		Size of Weight	
		Original	Augmented	Original	Augmented	Original	Augmented
Improved YOLOv4	416 × 416	72.5%	76.8%	68 h	89 h	282 MB	326 MB

**Table 6:** Comparison of fruit and vegetable classification models training and testing performance with the augmented dataset.

Models	Training Image Size	Training Results (AP50)	Testing Image Size	Testing Result (AP50)	Training Time	Iteration Number
YOLOv3 [45]	416 × 416	70.7%	608 × 608	67.7%	105 h	765
YOLOv3-tiny [45]		46.2%		44.5%	13 h	
YOLOv4 [11]		74.5%		72.6%	97 h	
YOLOv4-tiny [11]		53.6%		51.2%	10 h	
Parico et al. [36]		73.9%		71.8%	97 h	
Fu et al. [46]	224 × 224	67.4%		63.6%	68 h	
Liang et al. [47]	416 × 416	71.6%		68.4%	102 h	
Improved YOLOv4		75.8%		73.5%	92 h	

**Table 7:** Comparison of fruit and vegetable classification models average precision with the augmented dataset.

Model	Model Backbone	Image Size	AP	AP50	AP75	APS	APM	APL
YOLOv3 [45]	Darknet-53	608 × 608	41.7%	67.7%	42.4%	25.9%	43.2%	46.4%
YOLOv3-tiny [45]	Darknet-53		23.6%	44.5%	25.6%	14.3%	26.6%	32.7%
YOLOv4 [11]	CSPDarknet-53		49.3%	72.6%	55.2%	34.7%	54.8%	58.4%
YOLOv4-tiny [11]	CSPDarknet-53		28.5%	51.2%	32.5%	18.4%	33.5%	37.6%
Parico et al. [36]	CSPDarknet-53		48.7%	71.8%	55.1%	33.8%	52.7%	58.5%
Fu et al. [46]	-		36.2%	61.6%	37.4%	21.6%	38.4%	43.5%
Liang et al. [47]	Darknet-53		42.3%	68.4%	42.7%	24.7%	44.5%	47.3%
Improved YOLOv4	CSPDarknet-53		50.4%	73.5%	56.8%	33.5%	53.1%	60.3%

**Table 8:** Average frame processing time per sequence, measured in seconds. The average image input size is 640 by 640 pixels.

Image Processing and Transmission	Average Processing Time (s)
Image transmission using Bluetooth (between smart glass and smartphone)	0.054
Image transmission using 5G/Wi-Fi (between smartphone and server)	0.031
Image pre-processing	0.027
Fruit and vegetable classification	0.362
Fresh and rotten prediction	0.385
Total	0.859

## Data Availability

Dataset is available on https://www.kaggle.com/datasets/muhriddinmuxiddinov/fruits-and-vegetables-dataset (accessed on 10 September 2022).
